# Proteomic analysis of human synovial fluid reveals potential diagnostic biomarkers for ankylosing spondylitis

**DOI:** 10.1186/s12014-020-09281-y

**Published:** 2020-06-01

**Authors:** Ji-Hyun Lee, Jae Hun Jung, Jeesoo Kim, Won-Ki Baek, Jinseol Rhee, Tae-Hwan Kim, Sang-Hyon Kim, Kwang Pyo Kim, Chang-Nam Son, Jong-Seo Kim

**Affiliations:** 1 Division of Rheumatology, Department of Internal Medicine, School of Medicine, Keimyung University, Daegu, South Korea; 2 Department of Applied Chemistry, Institute of Natural Science, Global Center for Pharmaceutical Ingredient Materials, Kyung Hee University, Yongin, South Korea; 3 Center for RNA Research, Institute of Basic Science (IBS), Seoul, 08826 South Korea; 4 School of Biological Sciences, Seoul National University, Seoul, 08826 South Korea; 5 Department of Microbiology, School of Medicine, Keimyung University, Daegu, South Korea; 6 New Drug R&D Center, ARIBIO Co. Ltd., Seongnam, South Korea; 7 Department of Rheumatology, Hanyang University Hospital for Rheumatic Diseases, Seoul, South Korea

**Keywords:** Ankylosing spondylitis, Proteomics, Synovial fluid, Biomarker

## Abstract

**Background:**

Ankylosing spondylitis (AS) is a chronic inflammatory rheumatic disease affecting the axial skeleton and peripheral joints. The etiology of this disease remains poorly understood, but interactions between genetic and environmental factors have been implicated. The present study identified differentially expressed proteins in the synovial fluid (SF) of AS patients to elucidate the underlying cause of AS.

**Methods:**

A cohort of 40 SF samples from 10 AS and 10 each of rheumatoid arthritis (RA), gout, and osteoarthritis (OA) patients were analyzed by liquid chromatography tandem mass spectrometry (LC–MS/MS) to identify differentially expressed proteins specific to AS. The label-free LC–MS/MS results were verified by western blotting.

**Results:**

We identified 8 proteins that were > 1.5-fold upregulated in the SF of AS patients compared to that of the disease control groups, including HP, MMP1, MMP3, serum amyloid P-component (APCS), complement factor H-related protein 5 (CFHR5), mannose-binding lectin 2 (MBL2), complement component C9 (C9), and complement C4-A (C4A). CFHR5 and C9 were previously found in serum from AS patients, while APCS was previously found in SF as well as in serum. However, the present study has identified C4A, and MBL2 as potential AS biomarkers for the first time. The expression levels of MMP3, C9, and CFHR5 were verified in AS SF using western blotting.

**Conclusion:**

We performed quantitative comparative proteomic analysis using by LC–MS/MS of the SF from four disease states: RA, gout, and OA. This systematic comparison revealed novel differentially expressed proteins in AS SF, as well as two previously reported candidate biomarkers. We further verified the expression of MMP3, C9 and CFHR5 by western blot. These proteins may serve as diagnostic or prognostic biomarkers in patients with AS, and may thus improve the clinical outcomes of this serious disease.

## Background

Ankylosing spondylitis (AS) is a chronic inflammatory rheumatic disease, affecting the axial skeleton and peripheral joints, that occurs in 0.5% of the general population [1]. Without proper clinical treatment, AS causes permanent structural changes, leading to progressive disability that affects the quality of life [2]. While the exact etiology of this disease remains poorly understood, it may be caused by interactions between genetic and environmental factors [3]. AS represents an increasing financial burden on both patients and the healthcare system [4, 5].

AS is defined by the presence of definite structural changes in the sacroiliac joints. Although several possibilities for the etiology of AS, including human leukocyte antigen (HLA)-B27 and tumor necrosis factor-alpha (TNF-α), have been proposed as a therapeutic target [2, 4, 5], no clear cause for AS has been identified. Peripheral arthritis is an important feature of spondyloarthritis (SpA) in general [5, 6]. Swelling of the knees with inflammatory pain is a representative symptom of peripheral arthritis, and work-up for peripheral arthritis can identify all forms of SpA early and differentiate it from other arthritides. The synovial fluid (SF) from the knees may therefore contain diagnostic biomarkers for AS disease.

SF lubricates the joints, and consists of hyaluronic acid, inflammatory cells, and secreted proteins from the synovial fibroblasts, synovial membranes, and inflammatory cells [7]. Normally, SF is present in small amounts in all joints, acting as joint lubricant and providing nutrition for articular cartilage. However, when inflammation occurs, synovial cells secrete a large amount of synovial fluid and become the subjects of an inflammatory reaction that destroys the joints. Arthrocentesis is the extraction of synovial fluid and has been used for differential diagnosis in patients with inflammatory arthritis [8]. Although serologic and imaging techniques have been developed to diagnose rheumatic diseases, synovial fluid analysis is still used as an important diagnostic tool for differential diagnosis of arthritis, especially acute arthritic disease, since SF is in contact with the primary tissues affected by arthritic diseases and is implicated in the disease pathophysiology. Therefore, it is an excellent biofluid for the discovery of candidate biomarkers in arthritis-related diseases like AS [9].

In the last decade, many proteomic studies in the area of rheumatic diseases have been published [10–14], largely with the aim of diagnosing disease and evaluating disease activity/severity and therapeutic response [11, 12]. Different rheumatic disease samples with complicated pathologic structure, such as blood, SF, synovial tissue, and urine, have been investigated for system-wide discovery and validation of rheumatoid arthritis biomarkers [15]. Currently used diagnostic tests for rheumatoid arthritis (RA) include rheumatoid factor and anti-CCP antibodies (anti-cyclic citrullinated peptide antibodies, anti-CCP Ab) [16]. However, there is still no clinically available protein biomarker for early diagnosis and monitoring of AS. Although HLA-B27 is currently used for AS diagnosis, it has a high incidence of false positives. Although there were differences by race, the frequency of HLA-B27 was 4% in the normal control group and 83.3% in the patients with ankylosing spondylitis. Patients with negative HLA-B27 tend to be delayed in diagnosis because the symptoms are not typical. Therefore, the discovery of new robust biomarkers for AS is required for effective early diagnosis and treatment. In the present study, we performed a quantitative proteomics comparison of SF proteins isolated from AS patients [17] and three other arthritis patient groups (RA, gout, and OA). OA is a non-inflammatory arthritis, and was included as a disease control for inflammatory arthritic diseases [18], while both RA and gout were disease controls for inflammatory arthritis from different pathologic origins [19]. Following immunodepletion of super-abundant proteins and label-free quantitative proteomics, we found eight AS-specific and differentially expressed proteins in the AS group compared to the disease control groups. Further western blot verification confirmed the discriminatory ability of seven of these proteins in AS patients. Among these, MMP3 (Matrix metalloproteinase-3), C9 (complement component C9) and CFHR5 (Complement factor H-related protein 5) are upregulated in the SF of AS patients, and could therefore be potential biomarkers for AS diagnosis.

## Methods

### Subjects

Ten patients with AS were recruited from an outpatient rheumatology clinic in Hanyang University Hospital for Rheumatic Diseases, Seoul, South Korea. An additional 30 patients treated at Keimyung University Dongsan Hospital, Daegu, South Korea were recruited as disease control groups (10 OA, 10 gout, and 10 RA). These patient SF samples (first cohort) were used for the LC–MS/MS experiments and the associated western blot verification. We further recruited five more patient samples per each patient group (second cohort) for the independent western blot verification experiments (5 AS, 5 OA, 5 gout, and 5 RA). The patients met the 1984 New York criteria for ankylosing spondylitis [17] and the American College of Rheumatology criteria for RA [16], knee OA [18], and acute gout [19]. We collected demographic and clinical data from the subjects, including age, gender, disease duration, blood chemistry, and concomitant treatment. We provide the summary statistics (Table 1) and the original data including the diagnosis, clinical data, and current treatments of each patient enrolled in this study (Additional file 1: Table S1). The samples were obtained after getting informed consent from the patients. The study was approved by the ethical committee of the Keimyung University Dongsan Hospital (IRB 2015-12-022).

**Table 1:** The clinical and laboratory features of patients with ankylosing spondylitis, rheumatoid arthritis, gout, and osteoarthritis

Parameters	Ankylosing spondylitis	Rheumatoid arthritis	Gout	Osteoarthritis
No. of patients	10	10	10	10
Age (years)	32.6 ± 10.6	59.6 ± 11.6	62.8 ± 12.8	64.8 ± 8.5
Sex (M/F)	9/1	0/10	10/0	1/9
ESR (mm/h)	59.5 ± 39.7	32.7 ± 17.0	69 ± 21.3 (n = 5)	27.5 ± 15.2 (n = 4)
CRP (mg/dL)	4.9 ± 4.3	0.5 ± 3.7	3.4 ± 3.7 (n = 6)	0.1 ± 0.04 (n = 4)

CRP: C-reactive protein; ESR: erythrocyte sedimentation rate; F: female; M: male

### Synovial fluid sample collection and processing

Synovial fluid was collected by arthrocentesis in patients with knee joint pain and swelling. Contaminated bloods during arthrocentesis and samples where synovial fluids were generated for reasons other than the respective diseases were excluded. After centrifugation at 500×*g* for 10 min, five 1 mL vials of supernatant and 1 vial of sediment from each sample were stored at − 80 °C through the Keimyung University Dongsan Hospital Human Resource Bank.

### Immunodepletion of abundant proteins with MARS cartridge

We used a Multiple Affinity Removal System (MARS) Hu-14 spin cartridge (Agilent, 5188–6560), which contains a bulk of immobilized antibodies against the 14 most abundant proteins (albumin, IgG, antitrypsin, IgA, transferrin, haptoglobin, fibrinogen, alpha-2-macroglobulin, alpha-1-acid glycoprotein, IgM, apolipoprotein A1, apolipoprotein A2, complement C3 and transthyretin), which are known to occupy about 95% of plasma proteome [20], enabling immunodepletion of such abundant proteins in SF. The 0.22 μm membrane filter (Agilent) was used to remove particulates from the fluid samples by centrifugation at 100x*g* for 1.5 min. The flow-through was mixed with Buffer A LOAD/WASH (Agilent) and depleted according to the manufacturer’s instructions. During the depletion, flow-through was collected, and protein concentration was determined using a BCA assay. The column was routinely regenerated by eluting bound high-abundance proteins with buffer B and neutralizing with buffer A before further use. The acquired proteins were directly digested for total proteomic analysis.

### Peptide sample preparation

In-solution tryptic digestion and peptide cleanup were simultaneously performed in a 96-well plate for high reproducibility. Each depleted sample was supplemented with 8 M urea in 100 mM ammonium bicarbonate (ABC) (Sigma) and incubated at room temperature for 20 min. The samples were homogenized by vortexing and sonication twice. To each sample, dithiothreitol (Sigma) was added to be 10 mM for protein reduction at RT for 1 h. Then, iodoacetamide (Sigma) was added to be 30 mM for the cysteine alkylation at RT for 30 min in the dark. Samples were then diluted with 100 mM ABC prior to the addition of trypsin (MS grade, Pierce) at 1:50 of trypsin:sample ratio (w/w), and incubated at 37 °C for overnight. The trypsin was inactivated by acidification with 0.4% trifluoroacetic acid (Sigma). The acidified digests were immediately processed using a Sep-Pak C18 96-well plate (100 mg C18 sorbent per well, Waters). The peptides were eluted with 80% acetonitrile and then dried in a vacuum centrifuge.

### LC–MS/MS experiments

**Fig. 1:**
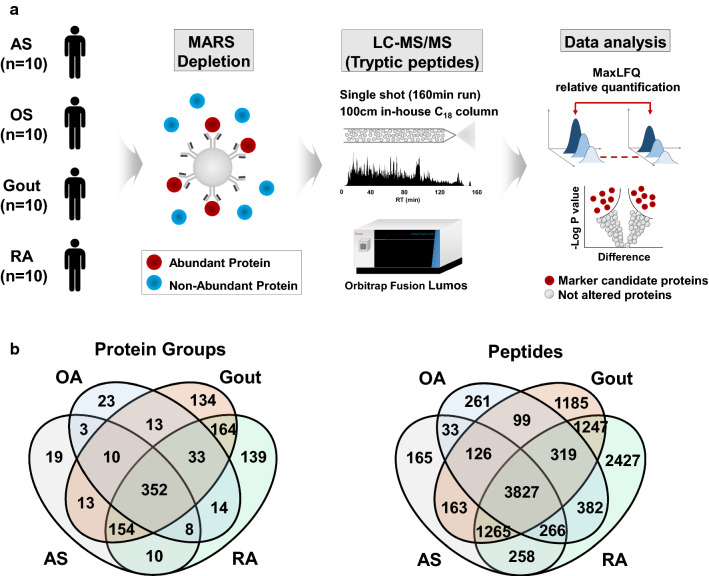
**a** Schematic diagram of proteomics experimental workflow. Synovial fluids of ankylosing spondylitis (AS), osteoarthritis (OA), gout, and rheumatoid arthritis (RA) were subject to immunodepletion using the multiple affinity removal system (MARS) and followed by trypsinization and LC–MS/MS analysis. MaxQuant LFQ (MaxLFQ) analysis was carried out for quantitative comparison. **b** Venn diagram showing the unique and shared protein groups and peptides identified in the synovial fluid of each group

We performed a label-free quantitative proteomics using LC–MS/MS experiments in synovial fluid samples. Forty LC–MS/MS experiments were carried out on an Orbitrap Fusion Lumos mass spectrometer (Thermo Fisher Scientific) coupled to a nanoACQUITY UPLC (Waters) with an in-house-packed trap (150 μm i.d. × 3 cm) and analytical column (75 μm i.d. × 100 cm) using 3 μm Jupiter C18 particles (Phenomenex). The LC gradient was as follows: from 5% to 40% solvent B (acetonitrile with 0.1% formic acid) for 130 min, then 40% to 80% solvent B for 5 min, holding at 80% solvent B for 10 min, and then equilibrating at 95% solvent A (water with 0.1% formic acid) for 30 min. Full MS data were acquired in a scan range of 375–1575 Th at a resolution of 60,000 at m/z 200, with an automated gain control (AGC) target value of 4.0 × 10^5^ and a maximum ion injection time of 50 ms. The maximal ion injection time for MS/MS was 50 ms at a resolution of 15,000 and an AGC target value of 5 × 10^4^. The dynamic exclusion time was set to 30 s. The resulting forty MS raw files were analyzed using MaxLFQ in Maxquant software (Fig. 1).

### Data analysis

MaxQuant (v.1.5.1.2) [21] was used to compare the acquired spectra to the UniProt human database (obtained in June 2018). Carbamidomethylation of cysteine was selected as a fixed modification, while N-terminal acetylation and methionine oxidation were set as variable modifications. 1% of false discovery rate (FDR) cutoff was applied at the levels of peptide-spectrum match and protein. An initial precursor mass deviation of up to 4.5 ppm and a fragment mass deviation of up to 20 ppm were allowed. To maximize the protein identification in initial discovery stage, the criterion with minimum stringency, at least one unique peptide, was applied for the protein identification. Proteins were quantified using the label-free quantification (LFQ) algorithm in MaxQuant [22]. The option of ‘match between runs’ was used for nonlinear retention time alignment. The match time window was 0.7 min, and the alignment time window was 20 min. Further statistical and bioinformatics analysis was performed using Perseus software (v.1.5.3.2) [23]. Proteins hit to the decoy database and contaminants, or proteins only identified by the peptide with modification sites such as methionine oxidation were filtered out prior to further analysis. A minimum of three valid protein quantification values across each clinical group was required for protein quantification. The LFQ intensity of individual proteins in the AS group was compared with that of such proteins in the other clinical groups following log2 transformation, i.e., AS vs OA, AS vs gout, and AS vs RA. Statistical analysis of the log2-transformed data was performed using two samples t-test (p value < 0.05). Proteins with fold change ≥ 1.5 or ≤ 0.67 between AS and other patient groups were considered to be differentially expressed proteins (DEPs) for AS group. The resulting DEPs were submitted to gene ontology (GO) annotation enrichment analysis.

### Enrichment and network analysis

To explore functional enrichment in the identified proteins in each of the clinical groups, GO analysis was performed using DAVID (version 6.8) [24]. We identified biological processes and KEGG pathways [25] that were enriched in our DEP list with p values of less than 0.05. To construct a network representing the enriched GO terms, we selected DEPs that are involved in the enriched cellular processes. We then built a protein network model using protein interaction information obtained from STRING version 11 database [26]. The interaction network models were visualized using Cytoscape [27].

### Western blot verification

We carried out western blot based verification experiments for the candidates as AS-specific proteins from LC–MS/MS proteomic data. To reduce the viscosity of SF, samples were treated with hyaluronidase (Sigma, H3884) at room temperature for 5 or 10 min, and then diluted 1:10 in RIPA buffer (Thermo Fisher) containing both protease and phosphatase inhibitor (Roche Diagnostics GmbH). The diluted sample was incubated on ice for five min, then transferred into new tube and centrifuged at 10,000×*g* at 4 °C for 15 min. Equal amounts of protein (30 μg) in each sample were aliquoted following protein quantitation by BCA assay (Thermo Fisher). Samples were mixed with sodium dodecyl sulfate (SDS) loading buffer and separated using SDS polyacrylamide gel electrophoresis (Western Blotting Kit, Hoefer Inc.). Proteins were then transferred onto nitrocellulose membranes (Amersham), blocked with 5% non-fat dried milk in tris-buffered saline containing 0.05% Tween-20 and immunoblotted with the appropriate primary and secondary antibodies. The antibodies used were as follows: rabbit polyclonal antibodies for C9 (1:5000, PA5-29093, Thermo Fisher), CFHR5 (1:500, ab175254, Abcam), MMP3 (1:5000, ab52915, Abcam), mannose-binding protein C (MBL2) (1:5000, ab189856, Abcam), complement C4-A (C4A) (1:5000, ab66790, Abcam), serum amyloid P-component (APCS) (1:5000, ab45151, Abcam), matrix metalloproteinase-1 (MMP1) (1:3000, ab38929, Abcam), anti-transferrin (1:5000, ab109503, Abcam), and peroxidase-conjugated AffiniPure Donkey anti-rabbit IgG (H + L) (1:10,000, Jackson Immunoresearch). Anti-transferrin (77 kDa) was used as a loading control [28].

## Results

### General characteristics of study subjects

The summary statistics and general characteristics of the study subjects are presented in Table 1 and Additional file 1: Table S1, respectively. The AS group was 32.6 (± 10.6) years of age, with 7.0 (± 4.6) years mean disease duration, 80% HLA-B27 positive rate, 20% biologics utilization, 59.5 (± 39.7) mm/h averaged ESR, and 4.9 (± 4.3) mg/dL averaged CRP. The RA group were all females at 59.6 (± 11.6) years of age, with 4.9 (± 4.9) years mean disease duration, 90% rheumatoid factor and anti-CCP antibody positive rate, 30% biologics utilization, 32.7 (± 17.0) mm/h averaged ESR, and 0.5 (± 3.7) mg/dL averaged CRP. The gout group were all males at 62.8 (± 12.8) years of age, with 0.3 (± 0.9) years of disease duration, and 7.1 (± 2.6) mg/dL concentration of serum uric acid. The OA group were 64.8 (± 8.5) years of age, with 5.5 (± 5.9) years mean disease duration.

### Proteomic analysis of SF from AS, RA, gout, and OA patient groups

**Fig. 2:**
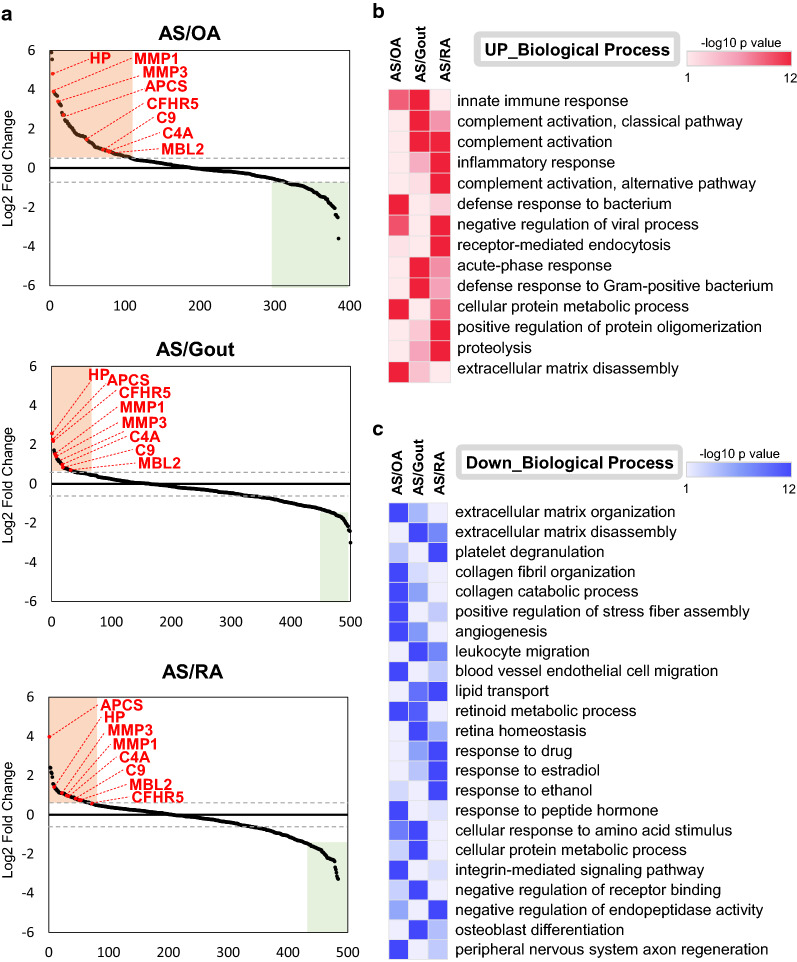
**a** Reverse cumulative plots of protein fold change in the synovial fluid from each disease group. The most commonly upregulated 8 proteins (HP, MMP1, MMP3, APCS, CFHR5, C9, C4A, and MBL2) are highlighted. **b**, **c** Gene ontology (GO) analysis of differentially expressed proteins in the synovial fluid of the AS group compared to in the OA, gout, and RA groups. Heatmaps showing significantly enriched GO biological process terms (p < 0.05) for differentially expressed proteins. The red color in the heatmap indicates a significant upregulation (**b**) and blue color indicates downregulation of the biological process (**c**)

To obtain insight into the molecular basis of AS in the SF, we performed a LFQ intensity-based proteomic comparison of SF from AS patients and SF from patients with OA, gout, and RA (Fig. 1a). All SFs were first subjected to immunodepletion of the 14 most abundant proteins using an MARS kit, and then followed by LC–MS/MS experiments, resulting in the identification of 569 proteins and 6103 peptides in the AS group, 874 proteins and 9991 peptides in the RA group, 456 proteins and 5313 peptides in the OA group, and 873 proteins and 8231 peptides in the gout group at < 1% FDR (Fig. 1b). A further MaxQuant analysis using ‘match between runs’ to minimize missing quantities between replicates and groups [21, 29] was then carried out. To resolve the AS-specific DEPs against other disease groups, we performed a series of pairwise statistical comparisons of AS group versus three different control groups (OA, gout and RA) based on the LFQ intensity of commonly identified proteins from ≥ 3 patients in each group using the Perseus software, resulting in 385, 500, and 485 quantifiable proteins for AS vs OA, AS vs gout, and AS vs RA comparison sets, respectively (Additional file 2: Table S2). DEPs in such three comparison sets were defined as the proteins with > 1.5 fold-change and < 0.05 *p* value and the corresponding numbers of up- and down-regulated DEPs were 102 and 84 for AS/OA set, 41 and 179 for AS/gout set, and 69 and 151 for AS/RA set, respectively (Additional file 3: Table S3). Intriguingly, we found that eight of the DEPs were AS-specifically up-regulated (HP, MMP1, MMP3, APCS, CFHR5, C9, C4A, and MBL2 in Table 2), whereas a total of 24 proteins including immune-related proteins such as PLA2G2A (Phospholipase A2) were commonly downregulated in AS samples against three other arthritides (Fig. 2a).

**Table 2:** List of proteins with increased levels in the synovial fluid of ankylosing spondylitis patient group vs other disease groups

Gene symbol	Protein	Fold change (AS/RA)	Fold change (AS/Gout)	Fold change (AS/OA)
HP	Haptoglobin	2.67	5.93	27.97
MMP3	Matrix metalloproteinase-3	2.02	2.63	10.13
CFHR5	Complement factor H-related protein 5	1.61	4.52	3.23
C9	Complement component C9	1.85	1.75	1.82
MBL2	Mannose-binding protein C	1.68	1.52	1.61
C4A	Complement C4-A	1.92	2.02	1.63
APCS	Serum amyloid P-component	15.69	4.81	6.53
MMP1	Matrix metalloproteinase-1	1.97	2.75	14.96

AS: ankylosing spondylitis; OA: osteoarthritis; RA: rheumatoid arthritis

### Gene ontology analysis to clarify the molecular basis for AS

Next, we tried to gain insight into the functional roles of the DEPs associated with AS (Fig. 2a and Table 2) via GO analysis of the up- and downregulated DEPs (Additional file 4: Table S4). Interestingly, the AS-specifically upregulated proteins showed enriched patterns largely to the immune response-related biological processes (Fig. 2b). In detail, the AS-specifically upregulated proteins vs OA or gout were strongly associated with the innate immune response (Fig. 2b). The enriched GO terms suggested that the AS-specifically upregulated proteins may play an important role in the complement activation and the inflammatory response. In addition, the GO terms involved in metabolic processes, protein oligomerization, proteolysis, and extracellular matrix disassembly were also enriched in the AS-specific DEPs. On the contrary, the GO terms related to various biological processes including extracellular matrix organization, collagen fibril organization, and cellular structure were enriched in the AS-specifically downregulated DEPs (Fig. 2c).

### Protein–protein interaction network describing AS

**Fig. 3:**
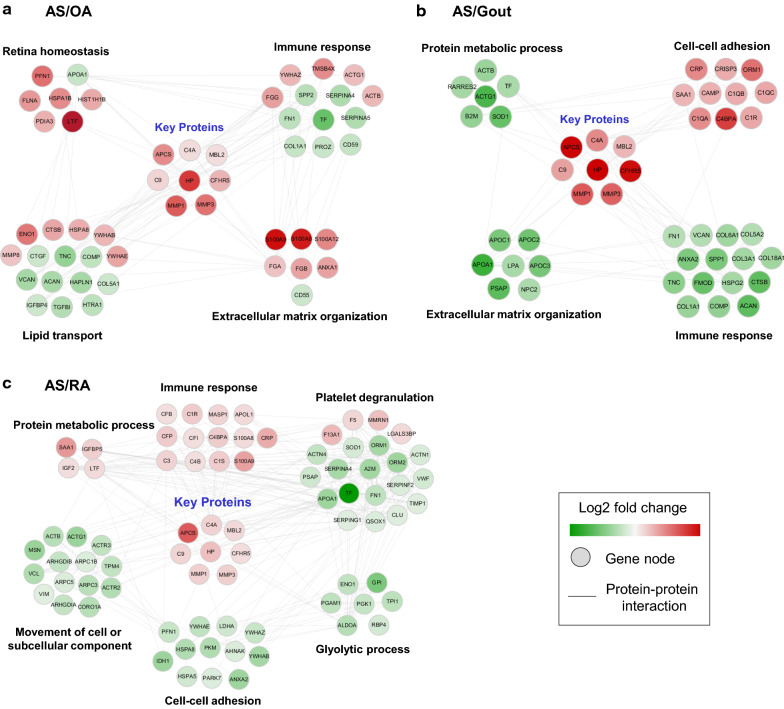
Network modelling of differentially expressed proteins in the synovial fluid of the AS group versus the **a** OA, **b** gout and **c** RA group. Protein–protein interaction network showing the biological processes affected, including the immune response, platelet degranulation, cell–cell adhesion etc. The colors of the nodes represent proteins with increased (red) or decreased (green) levels in the synovial fluid of the AS group compared to in the other groups. The connection between nodes (solid grey lines) shows either a regulatory role or physical interaction between proteins

To better understand the cellular networks that are altered in the synovial fluid of AS patients, we created protein–protein interaction network models of AS-specific upregulated DEPs using the STRING database (Fig. 3). Four important processes were enriched in the AS-specific upregulated DEPs: innate immune response, complement activation, platelet degranulation, and glycolytic process. We found that APCS (innate immune response), C9, C4A, CFHR5, and MBL2 (complement activation), which are common in AS SF, are known to interact based on this network.

### Verification by western blot analysis

**Fig. 4:**
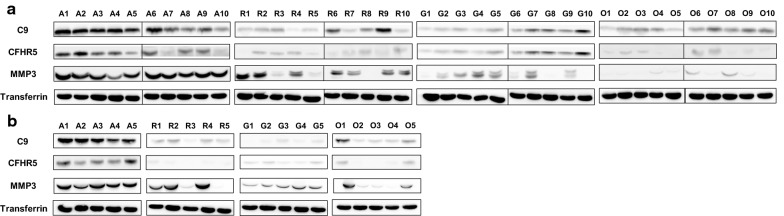
Verification of C9, CFHR5, and MMP3 in synovial fluid by western blot. **a** Western blot analysis in the original synovial fluid sample set: A; AS (n = 10), R; RA (n = 10), G; gout (n = 10), and O; OA (n = 10). **b** Western blot analysis in the independent sample set: AS (n = 5), RA (n = 5), gout (n = 5), OA(n = 5). Transferrin was used as an input amount control

Following the LC–MS/MS analysis, the levels of 7 DEPs in SF (MMP3 (50 kDa), CFHR5 (70 kDa), C9 (60 kDa), MBL2 (26 kDa), C4A (95 kDa), APCS (25 kDa), and MMP1 (50 kDa), except for HP, which occupies a large portion in synovial fluid) were verified by western blot using specific primary antibodies. Figure 4 clearly showed that the three candidates (MMP3, CFHR5, and C9) are reproducibly represented as AS-specific proteins in both original (Fig. 4a) and additional independent (Fig. 4b) sample sets, further verifying the results from mass spectrometry. MMP3 was considered as a positive control because it has been known to be highly expressed in the SF of AS patients [30], expectedly showing the significantly increased expression levels in AS SF compared to those in the other clinical groups. MMP1 protein was excluded due to the vain western blot detection and transferrin protein was included in western blot experiments as a control of loading amount.

However, the AS-specifically upregulated expressions of the remaining three proteins (C4A, MBL2 and APCS) in mass spectrometry were not clearly verified by the original western blot experiments (Additional file 5: Figure 1a). Intriguingly, C4A protein showed a clear AS-specific pattern in the independent sample set (Additional file 5: Figure 1b), suggesting that the C4A protein needs to be further verified or included to the validation candidates of AS-specific markers.

## Discussion

Early diagnosis of AS is difficult because the etiology is not clear and there is no specific diagnostic indicator [31]. Mass spectrometry-based proteomics is a state-of-the-art analytical technique that enables the discovery of indicator proteins for the diagnosis and treatment of diseases. There has been a recent expansion in proteomics research on a number of different rheumatic diseases [32].

In the present study, we performed comprehensive proteomic profiling and western blot analysis of SF obtained from patients with AS and SF obtained from patients with comparative diseases (RA, gout, and OA). SF samples obtained from patients with AS and the three disease control groups were quantified using highly sensitive LC–MS/MS and LFQ-based analysis. We discovered eight biomarker candidate proteins (HP, MMP3, CFHR5, C9, MBL2, C4A, APCS, and MMP1) with > 1.5 of fold change in AS compared to that in the other groups. Western blot experiments for seven proteins (with the exception of HP) were performed to further verify the differential expression level. As a result, three proteins (MMP3, CFHR5 and C9) were solidly verified to be highly expressed in the SF of patients with AS.

MMP family proteins are involved in the pathogenesis of arthritis. In particular, MMP3 is a protease that is synthesized and secreted by fibroblasts and chondrocytes in synovial joints, and also activates other MMPs such as MMP1, MMP7, and MMP9 [33]. A recent meta-analysis suggests that the serum levels of MMP3 rise in AS patients [29]. The validity of our results was further enhanced by the discovery of previously researched AS relevant protein, MMP3.

CFHR5 plays an important role in the alternative pathway complement system, and binds to C3 to activate it [34]. This protein is highly expressed in the serum of AS patients [35]. In addition, high concentrations of CFHR5 were observed in the plasma of patients with systemic lupus erythematosus, an autoimmune disease [36]. C9 is a member of the membrane attack complex (MAC) complement system, and causes lysis by inducing pores in the cell membrane after activating C5, the final stage of the complement system [37]. The levels of this protein are elevated in the serum of AS patients [35].

Unlike the proteomic result, MBL2 showed inconsistently and slightly upregulated expression level according to the western blotting results. The expression of MBL2 was observed in 11 out of 15 cases of AS in the western blot, compared to six in RA, six in gout, and three in degenerative arthritis. MBL2 has an ability to recognize carbohydrate types found on pathogenic microbial surfaces. MBL2 initiates the lectin pathway and opsonizes apoptotic and necrotic cells [38].

C4A, a complementary protein with MBL2, showed vague expression pattern in the original western blot set, while a clear AS-specific pattern in the independent western blot set. C4A is involved in the classical pathway of the complement system. Deficiency of C4A is associated with systemic lupus erythematosus and type 1 diabetes mellitus, and its overexpression is associated with mental disorders such as schizophrenia and bipolar disorder [39]. The complement system is an important mechanism of humoral and innate immunity. Suppressing the complement system in an animal model of AS may improve AS treatment [40]. The complement system is active in patients with systemic sclerosis, a musculoskeletal disorder [41].

APCS is associated with the innate immune system and is known to have increased expression in the SF and serum of AS patients [35, 42]. However, APCS showed quite consistent expression patterns for all SF samples in western blot data, even though MS-based proteomics revealed significantly higher fold-changes of APCS protein in the AS group than the other groups (AS/RA: 15.69, AS/gout: 4.81, AS/OA: 6.53) (Table 2). Previously, CRP and APCS were shown to have 51% sequence homology in rats [43]. In this regard, the antibody used in this study might not be exclusively specific to APCS, simultaneously detecting other inflammatory markers too. The antibody-based verification approach may therefore not be suitable in all cases, and an alternative approach may be better for this particular protein. For instance, targeted proteomics such as multiple reaction monitoring can be an alternative. The last candidate, MMP1, a protein mainly expressed in bone metabolism, was not detected by western blot in all disease groups, and should be verified further with other approach.

The limitations of the present study are the following: the relatively small number of patients included due to the difficulty in obtaining clinical sample donor and the lack of further validation method beyond western blotting. Further validation will increase the likelihood of identifying biomarkers for AS, as will increasing the number of samples and using alternative sample types including urine, serum, synovial membrane, and animal models.

## Conclusions

In total, 1089 proteins were identified by label-free comparative proteomic analysis in the SF of patients with AS, RA, gout, and OA. This is the largest dataset of proteins identified in the synovial fluid to date. This is also the first time that three diseases (RA, gout, and OA) have been used simultaneously as disease control groups during proteomic profiling of AS. Out of the significantly dysregulated proteins, eight were significantly increased in the SF of AS patients compared to that of patients of the other three diseases. Out of these, MBL2 and C4A were the first proteins previously reported as AS markers, while the remaining six proteins were first reported as AS markers in SF sample in this study. Four out of the eight proteins are part of the complement system, which appears to be highly associated with AS. Altogether, these results suggest an important role for complement signaling during AS disease progression, and this avenue of enquiry may provide insight into the underlying molecular mechanisms of AS. The clinical utility of the putative biomarkers identified in the present study should be further validated in a larger cohort.

## Supplementary information

**Additional file 1: Table S1.** Baseline characteristics of the first patient cohort used for the LC-MS/MS experiments and the associated western blot verification (a), and the second patient cohort used for the western blot verification (b).

**Additional file 2: Table S2.** Quantifiable protein list of AS patient group versus three different control groups (OA, gout and RA), showing 385, 500, and 485 quantifiable proteins for AS vs OA, AS vs gout, and AS vs RA comparison sets, respectively. Protein accession number, gene symbols, fold-change, and *p*-value are provided.

**Additional file 3: Table S3.** Differentially expressed proteins (DEPs) in AS vs OA, AS vs gout, and AS vs RA comparison sets, defined as the proteins with > 1.5-fold-change and < 0.05 *p*-value. Gene symbols, Log2 fold-change and descriptions of the proteins are provided.

**Additional file 4: Table S4.** Gene ontology analysis in terms of biological process performed by DAVID tool for the up-regulated (a–c) or and down-regulated DEPs (d–f) in OA, Gout, and RA groups, versus AS group, respectively.

**Additional file 5: Figure** **S1**. Verification of C4A, MBL2, and APCS in synovial fluid by western blot. (a) Western blot analysis in the original synovial fluid sample set: A; AS (n = 10), R; RA (n = 10), G; gout (n = 10), and O; OA (n = 10). (b) Western blot analysis in the Independent sample set: AS (n = 5), RA (n = 5), gout (n = 5), OA(n = 5). Transferrin was used as an input amount control.

## Data Availability

All of the raw data has been deposited into the PRIDE Archive (ProteomeXchange) with the data set identifier PXD016620.

## Abbreviations

AS: Ankylosing spondylitis
SF: Synovial fluid
OA: Osteoarthritis
RA: Rheumatoid arthritis
HLA: Human leukocyte antigen
TNF: Tumor necrosis factor
CCP: Cyclic citrullinated peptide
AGC: Automated gain control
FDR: False discovery rate
LFQ: Label-free quantification
DEP: Differentially expressed proteins
SDS: Sodium dodecyl sulfate
bDMARD: Biologic disease-modifying anti-rheumatic drug
cDMARD: Conventional disease-modifying anti-rheumatic drug
CRP: C-reactive protein
ETN: Etanercept
ESR: Erythrocyte sedimentation rate
HP: Haptoglobin
MMP1: Matrix metalloproteinase-1
MMP3: Matrix metalloproteinase-1
APCS: Serum amyloid P-component
CFHR5: Complement factor H-related protein 5
C9: Complement component C9
C4A: Complement C4-A
MBL2: Mannose-binding protein C
MAC: Membrane attack complex
