# Correction to: Proteomic analysis of human synovial fluid reveals potential diagnostic biomarkers for ankylosing spondylitis

**DOI:** 10.1186/s12014-023-09423-y

**Published:** 2023-09-02

**Authors:** Ji-Hyun Lee, Jae Hun Jung, Jeesoo Kim, Won-Ki Baek, Jinseol Rhee, Tae-Hwan Kim, Sang-Hyon Kim, Kwang Pyo Kim, Chang-Nam Son, Jong-Seo Kim

**Affiliations:** 1 Division of Rheumatology, Department of Internal Medicine, School of Medicine, Keimyung University, Daegu, South Korea; 2 Department of Applied Chemistry, Institute of Natural Science, Global Center for Pharmaceutical Ingredient Materials, Kyung Hee University, Yongin, South Korea; 3 Center for RNA Research, Institute of Basic Science (IBS), Seoul, 08826 South Korea; 4 School of Biological Sciences, Seoul National University, Seoul, 08826 Korea; 5 Department of Microbiology, School of Medicine, Keimyung University, Daegu, South Korea; 6 New drug R&D Center, ARIBIO Co. Ltd, Seongnam, South Korea; 7 Department of Rheumatology, Hanyang University Hospital for Rheumatic Diseases, Seoul, South Korea

Following publication of the original article [1], the authors noticed the errors in Fig. 4 and Supplementary Fig. 1.

In the Fig. 4, the CFHR5 band of G1-10 is duplicated with the C9 band.

In the Fig. 4 and Supplementary Fig. 1, the transferrin band of O6-10 is duplicated with the A6-10, independent sample O1-5.

In the Fig. 4 and Supplementary Fig. 1, the transferrin band of R6-10 is duplicated with the independent sample G1-5.

The corrected version of Fig. 4 is given below. The corrected supplementary file 5 is provided in this Correction article. The corrected figures do not alter the overall conclusions of this study, and all other data still stand. The authors regret this error and apologize for any confusion or inconvenience it may have caused.

**Fig. 4:**
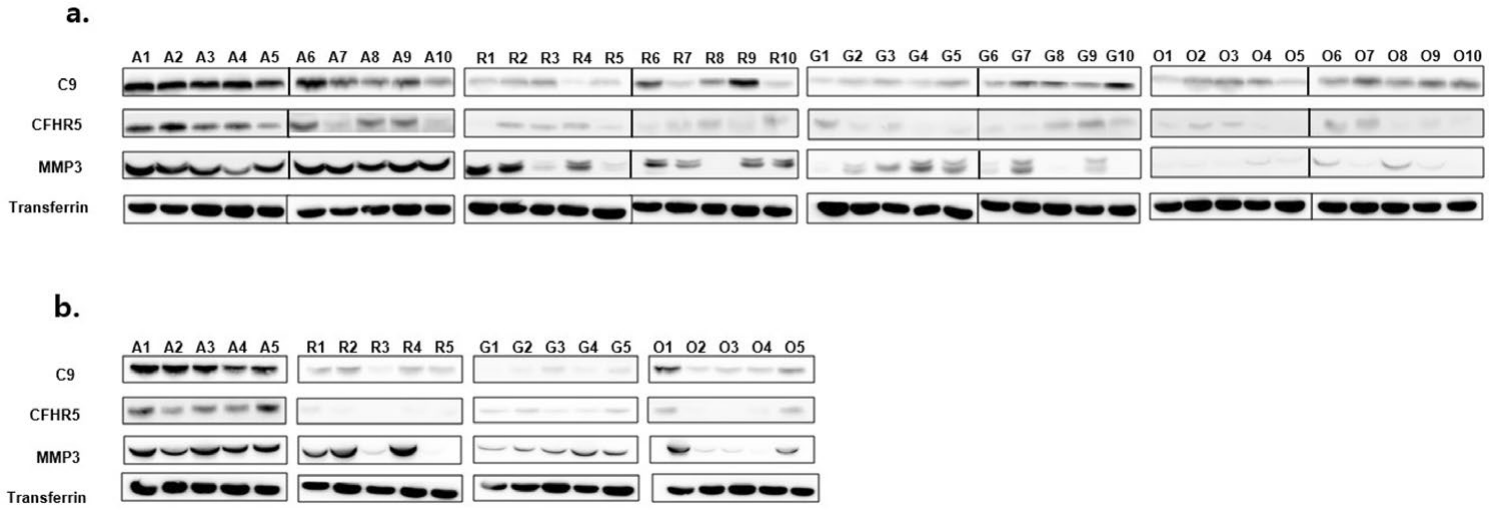
Verification of C9, CFHR5, and MMP3 in synovial fluid by western blot. **a** Western blot analysis in the original synovial fluid sample set: A; AS (n = 10), R; RA (n = 10), G; gout (n = 10), and O; OA (n = 10). **b** Western blot analysis in the independent sample set: AS (n = 5), RA (n = 5), gout (n = 5), OA (n = 5). Transferrin was used as an input amount control

### Electronic supplementary material

Below is the link to the electronic supplementary material.

Supplementary Material 1 S1. Verification of C4A, MBL2, and APCS in synovial fluid by western blot. (a) Western blot analysis in the original synovial fluid sample set: A; AS (n = 10), R; RA (n = 10), G; gout (n = 10), and O; OA (n = 10). (b) Western blot analysis in the Independent sample set: AS (n = 5), RA (n = 5), gout (n = 5), OA(n = 5). Transferrin was used as an input amount control.
