# Supplementary material for: Proteomic analysis of human synovial fluid reveals potential diagnostic biomarkers for ankylosing spondylitis
Source: Clin Proteomics. 2020 Jun 1;17:20. doi: 10.1186/s12014-020-09281-y (PMC7269004; doi:10.1186/s12014-020-09281-y)
Supplement: Supplementary file 1 — Additional file 1: Table S1. Baseline characteristics of the first patient cohort used for the LC-MS/MS experiments and the associated western blot verification (a), and the second patient cohort used for the western blot verification (b). [file 12014_2020_9281_MOESM1_ESM.pdf]

Supplementary Tables S1. Baseline characteristics of two sets of cohorts.

(a) Baseline characteristics of first patient cohort used for the LC-MS/MS experiments and the associated western blot verification

Diseases/Number	Gender	Age	ESR (mm/hr)	CRP (mg/dL)	HLA-B27	RF (ng/mL)	Anti-CCP (U/mL)	Serum UA (mg/dL)	Disease Duration (yr)	bDMARD	cDMARD
A1	M	39	81	5.1	Positive	NA	NA	NA	2	IFX	None
A2	M	41	127	15.8	Positive	NA	NA	NA	10	GLM	None
A3	M	47	46	4.8	Negative	NA	NA	NA	3	None	MTX+SSZ
A4	M	21	7	8	Positive	NA	NA	NA	6	None	None
A5	M	24	53	4.7	Positive	NA	NA	NA	7	None	SSZ
A6	M	35	27	2.6	Positive	NA	NA	NA	17	None	MTX+SSZ
A7	M	34	79	2	Positive	NA	NA	NA	5	None	SSZ
A8	M	45	114	2.3	Positive	NA	NA	NA	11	None	SSZ
A9	M	22	39	3.2	Positive	NA	NA	NA	9	None	SSZ
A10	F	18	22	0.7	Negative	9.3	0	NA	0	None	None
R1	F	57	64	1.4	NA	47.4	>200	NA	0.3	None	MTX+SSZ
R2	F	47	25	1.0	NA	92	>200	NA	0.5	None	MTX+SSZ
R3	F	42	45	0.5	NA	39.5	>200	NA	5	None	MTX
R4	F	51	2	0.0	NA	785.4	>200	NA	3	TCZ	MTX
R5	F	69	47	0.0	NA	35.7	129.5	NA	5	IFX	MTX
R6	F	62	26	0.6	NA	310	>200	NA	5	None	MTX+LEF
R7	F	53	39	0.5	NA	46.3	>200	NA	8	None	MTX
R8	F	64	33	0.1	NA	99.7	16.6	NA	17	None	HCQ
R9	F	76	20	0.9	NA	9.3	0	NA	1	None	MTX
R10	F	75	26	0.0	NA	133	>200	NA	4	ETN	MTX
G1	M	73	NA	0.5	NA	NA	NA	8.4	0	None	None
G2	M	71	105	1.8	NA	9.3	0.6	9	0	None	None
G3	M	47	NA	2.3	NA	NA	NA	7.5	0	None	None
G4	M	62	68	1.6	NA	NA	NA	8.6	0	None	None
G5	M	42	66	4.5	NA	22	1.9	6.9	0	None	None

G6	M	63	NA	NA	NA	9.3	NA	9.9	0.2	None	None
G7	M	51	NA	NA	NA	NA	NA	6.8	0.1	None	None
G8	M	63	NA	NA	NA	NA	NA	9	3	None	None
G9	M	82	54	1.9	NA	11.4	NA	3.5	0	None	None
G10	M	74	52	11.4	NA	9.3	0	1.5	0	None	None
O1	F	70	30	0.1	NA	9.3	NA	NA	4	None	None
O2	F	53	NA	NA	NA	9.3	0.6	NA	6	None	None
O3	F	64	48	0.1	NA	NA	NA	NA	4	None	None
O4	F	72	17	0.1	NA	10.5	0	NA	0	None	None
O5	F	63	NA	NA	NA	9.3	1.1	NA	2	None	None
O6	M	64	NA	NA	NA	9.3	NA	NA	6	None	None
O7	F	57	NA	NA	NA	NA	NA	NA	17	None	None
O8	F	72	NA	NA	NA	NA	NA	NA	1	None	None
O9	F	79	15	0.03	NA	9.3	0.9	NA	0	None	None
O10	F	54	NA	NA	NA	NA	NA	NA	15	None	None

Anti-CCP: anti-cyclic citrullinated peptide antibodies; bDMARD: biologic disease-modifying anti-rheumatic drug; cDMARD: conventional disease-modifying anti-rheumatic drug; CRP: C-reactive protein; ETN: etanercept; ESR: erythrocyte sedimentation rate; F: female; G: gout; GLM: golimumab; HCQ: hydroxychloroquine; IFX: infliximab; LEF: leflunomide; M: male; MTX: methotrexate; NA: not available; RF: rheumatoid factor; SSZ: sulfasalazine; TCZ: tocilizumab; UA: uric acid

(b) Baseline characteristics of second patient cohort used for the western blot verification

Diseases/Number	Gender	Age	ESR (mm/hr)	CRP (mg/dL)	HLA-B27	RF (ng/mL)	Anti-CCP (U/mL)	Serum UA (mg/dL)	Disease Duration (yr)	bDMARD	cDMARD
A’1	F	54	102	4.65	Positive	NA	NA	3.4	1	None	None
A’2	M	51	69	3.79	Positive	NA	NA	4.7	12	None	None
A’3	M	34	66	10.65	Positive	NA	NA	6.1	4	None	SSZ
A’4	M	51	69	3.79	Positive	NA	NA	4.7	12	None	None
A’5	M	36	112	10.24	Positive	NA	NA	4.5	0.1	None	None
R’1	F	70	106	2.23	NA	29.8	198.5	NA	0	None	None
R’2	F	32	35	1.97	NA	9.3	NA	NA	0	None	None
R’3	F	80	NA	NA	NA	NA	NA	NA	5.8	None	None
R’4	F	22	26	0.15	NA	149.8	7.1	NA	0	None	None
R’5	M	81	94	0.01	NA	177.8	>200.0	NA	1.4	None	HCQ
G’1	F	68	NA	NA	NA	NA	NA	3	4.3	None	None
G’2	M	45	47	17.76	NA	NA	NA	10.9	0	None	None
G’3	M	62	68	1.6	NA	NA	NA	8.6	0.1	None	None
G’4	M	35	51	7.56	NA	NA	NA	2.6	0.25	None	None
G’5	M	81	120	5.6	NA	NA	NA	4	0	None	None
O’1	F	51	NA	NA	NA	NA	NA	NA	0	None	None
O’2	F	73	NA	NA	NA	NA	NA	NA	3.1	None	None
O’3	F	67	NA	NA	NA	NA	NA	NA	0.3	None	None
O’4	F	77	NA	NA	NA	NA	NA	NA	0.3	None	None
O’5	M	58	NA	NA	NA	NA	NA	NA	0	None	None

Anti-CCP: anti-cyclic citrullinated peptide antibodies; bDMARD: biologic disease-modifying anti-rheumatic drug; cDMARD: conventional disease-modifying anti-rheumatic drug; CRP: C-reactive protein; ETN: etanercept; ESR: erythrocyte sedimentation rate; F: female; G: gout; GLM: golimumab; HCQ: hydroxychloroquine; IFX: infliximab; LEF: leflunomide; M: male; MTX: methotrexate; NA: not available; RF: rheumatoid factor; SSZ: sulfasalazine; TCZ: tocilizumab; UA: uric acid
