# Supplementary material for: Proteomic analysis of human synovial fluid reveals potential diagnostic biomarkers for ankylosing spondylitis
Source: Clin Proteomics. 2020 Jun 1;17:20. doi: 10.1186/s12014-020-09281-y (PMC7269004; doi:10.1186/s12014-020-09281-y)
Supplement: Supplementary file 5 — Additional file 5: Figure S1. Verification of C4A, MBL2, and APCS in synovial fluid by western blot. (a) Western blot analysis in the original synovial fluid sample set: A; AS (n = 10), R; RA (n = 10), G; gout (n = 10), and O; OA (n = 10). (b) Western blot analysis in the Independent sample set: AS (n = 5), RA (n = 5), gout (n = 5), OA(n = 5). Transferrin was used as an input amount control. [file 12014_2020_9281_MOESM5_ESM.pdf]

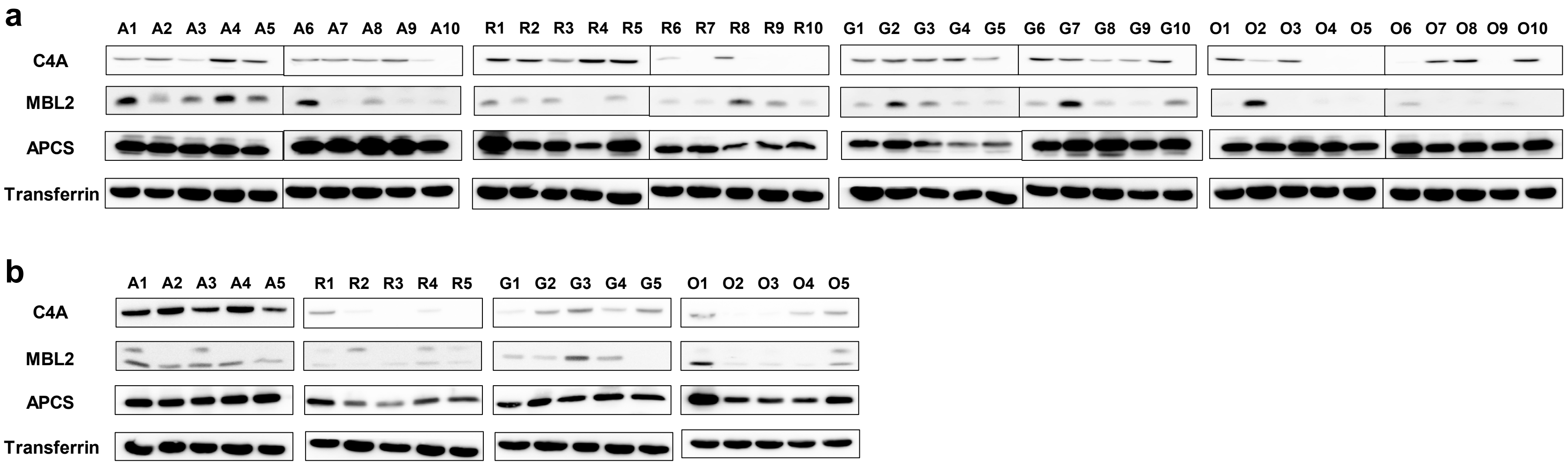

Supplementary Figure 1. Verification of C4A, MBL2, and APCS in synovial fluid by western blot. (a) Western blot analysis in the original synovial fluid sample set: A; AS (n=10), R; RA (n=10), G; gout (n=10), and O; OA (n=10). (b) Western blot analysis in the Independent sample set: AS (n=5), RA (n=5), gout (n=5), OA(n=5). Transferrin was used as an input amount control.
