# Supplementary material for: Correction to: Proteomic analysis of human synovial fluid reveals potential diagnostic biomarkers for ankylosing spondylitis
Source: Clin Proteomics. 2023 Sep 2;20:34. doi: 10.1186/s12014-023-09423-y (PMC10474741; doi:10.1186/s12014-023-09423-y)
Supplement: Supplementary file 1 — Supplementary Material 1 S1. Verification of C4A, MBL2, and APCS in synovial fluid by western blot. (a) Western blot analysis in the original synovial fluid sample set: A; AS (n = 10), R; RA (n = 10), G; gout (n = 10), and O; OA (n = 10). (b) Western blot analysis in the Independent sample set: AS (n = 5), RA (n = 5), gout (n = 5), OA(n = 5). Transferrin was used as an input amount control. [file 12014_2023_9423_MOESM1_ESM.docx]

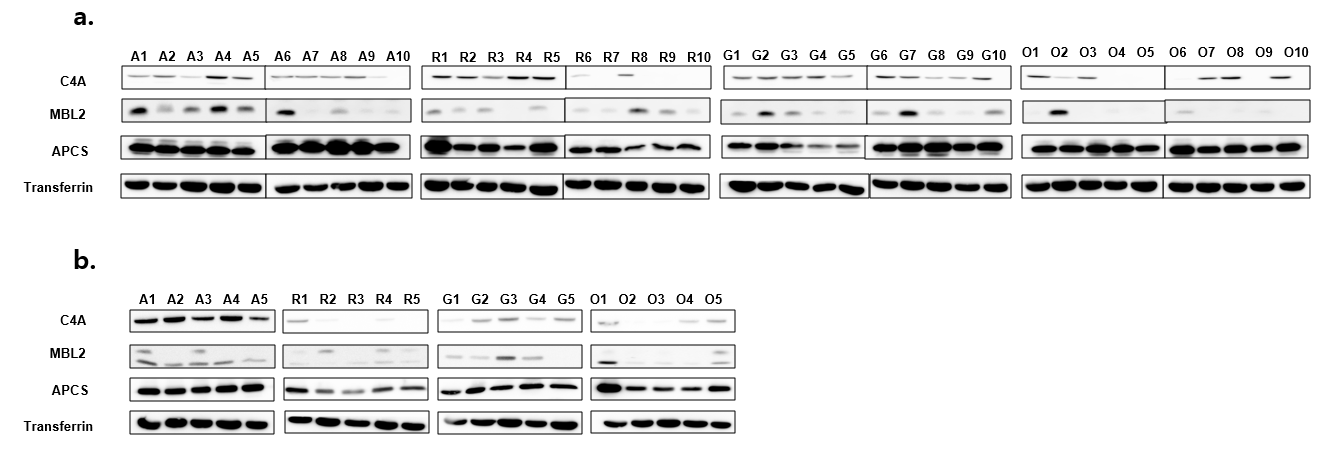


**Supplementary Figure S1**. Verification of C4A, MBL2, and APCS in synovial fluid by western blot. (a) Western blot analysis in the original synovial fluid sample set: A; AS (n=10), R; RA (n=10), G; gout (n=10), and O; OA (n=10). (b) Western blot analysis in the Independent sample set: AS (n=5), RA (n=5), gout (n=5), OA(n=5). Transferrin was used as an input amount control.
